# How Well Do Health-Mediation Programs Address the Determinants of the Poor Health Status of Roma? A Longitudinal Case Study

**DOI:** 10.3390/ijerph14121569

**Published:** 2017-12-13

**Authors:** Andrej Belak, Zuzana Dankulincova Veselska, Andrea Madarasova Geckova, Jitse P. van Dijk, Sijmen A. Reijneveld

**Affiliations:** 1 Department of Health Psychology, Faculty of Medicine, P. J. Safarik University, 04011 Košice, Slovakia; zuzana.dankulincova@upjs.sk (Z.D.V.); andrea.geckova@upjs.sk (A.M.G.); 2 Kosice Institute for Society and Health, Faculty of Medicine, P. J. Safarik University, 04011 Košice, Slovakia; j.p.van.dijk@umcg.nl; 3 Department of Community and Occupational Medicine, University Medical Center Groningen, University of Groningen, 9713 AV Groningen, The Netherlands; s.a.reijneveld@umcg.nl; 4 Department of General Anthropology, Faculty of Humanities, Charles University, 158 00 Prague, Czech Republic; 5 Olomouc University Society and Health Institute, Palacky University, 771 11 Olomouc, Czech Republic

**Keywords:** Slovakia, Roma, health inequities, ethnicity, social determinants, policy evaluation, qualitative research, community health

## Abstract

In Central and Eastern Europe (CEE), health-mediation programs (HMPs) have become central policy instruments targeting health inequities between segregated Roma and general populations. Social determinants of health (SDH) represent the root causes behind health inequities. We therefore evaluated how an HMP based in Slovakia addressed known SDH in its agenda and its everyday implementation. To produce descriptive data on the HMP’s agenda and everyday implementation we observed and consulted 70 program participants across organizational levels and 30 program recipients over the long-term. We used a World Health Organization framework on SDH to direct data acquisition and consequent data content analysis, to structure the reporting of results, and to evaluate the program’s merits. In its agenda, the HMP did not address most known SDH, except for healthcare access and health-related behaviours. In the HMP’s everyday implementation, healthcare access facilitation activities were well received, performed as set out and effective. The opposite was true for most educational activities targeting health-related behaviours. The HMP fieldworkers were proactive and sometimes effective at addressing most other SDH domains beyond the HMP agenda, especially material conditions and psychosocial factors, but also selected local structural aspects. The HMP leaders supported such deliberate engagement only informally, considering the program inappropriate by definition and too unstable institutionally to handle such extensions. Reports indicate that the situation in other CEE HMPs is similar. To increase the HMPs’ impact on SDH, their theories and procedures should be adapted according to the programs’ more promising actual practice regarding SDH.

## 1. Introduction

In Central and Eastern Europe (CEE), facing and adapting to a long and ongoing history of prejudice, unfair treatment and paternalist remedial policies towards the Roma [1,2,3,4,5] segregated Roma communities systematically occupy the lowest societal positions according to socioeconomic and health-status measures. Compared with the general populations, they attain the lowest levels of education and income and have the highest rates of unemployment [6,7]. At the same time, they have the shortest life-spans, while facing the greatest burdens of both infectious and non-communicable diseases [8,9,10].

Over the last decade, health-mediation programs (HMPs) have increasingly become adopted by CEE state administrations as their main policy instruments targeting the health inequities between segregated Roma and the respective general populations. Modelled on analogous services in Western Europe, most HMPs originally consisted primarily of community health workers facilitating individual healthcare access [11,12,13,14]. In countries with the longest health-mediation traditions (Romania, Bulgaria and Slovakia), the initial small-scale projects, predominantly driven by non-governmental organizations (NGOs), have eventually been transformed into more complex, state-backed national-level programs. The countries’ officials increasingly present HMPs as central state policies for tackling health inequities between Roma and non-Roma [8,14,15].

**Figure 1:**
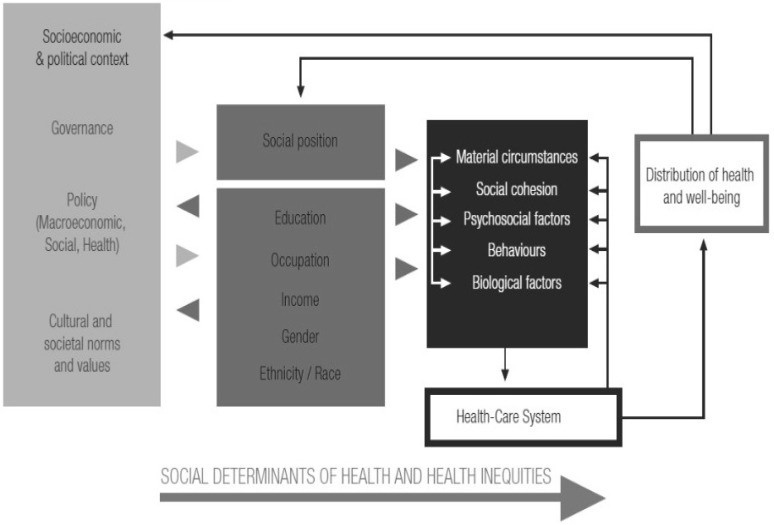
The World Health Organization’s (WHO) Conceptual framework for action on social determinants of health (amended from: [18]). The three columns left from the ‘Distribution of health and well-being’ box represent the three major categories of SDH, each defined through the particular determinants it lists. From left to right, the categories are: ‘Socio-political context’, ‘Structural SDH’ and (optionally including healthcare system related determinants represented by the so-named box) ‘Intermediary SDH’ (for further details, see also the original WHO source: [18]).

According to contemporary public health theory [16,17,18,19], many other social determinants of health (SDH) need to be accounted for beyond healthcare access to alleviate existing health inequities. Socio-epidemiological evidence over the last 50 years has shown that major health inequities result *primarily* from systematic disparities in the conditions that contribute to people becoming sick, rather than from systematic differences in healthcare access among the people already sick [16,17,18,19]. Consequently, to tackle such inequities we both need interventions focused on gaps in access to healthcare and interventions aimed at conditions which contribute to people becoming sick. Drawing on the same body of evidence, such interventions should address various conditions that cross the traditional bio/psycho/social divides [19]. For instance, a widely used World Health Organization’s Framework for action on SDH (WHO SDH Framework) [18] includes and distinguishes three levels of factors. The first is local exposures and vulnerabilities, i.e., ‘intermediary determinants’, which regard material conditions, social cohesion, psychosocial factors, lifestyle behaviours and biological factors. The second regards more indirect socially driven circumstances affecting these intermediary factors, i.e., ‘structural determinants’, comprising educational, occupational, income, gender and racial/ethnic statuses. And the third level regards the ‘sociopolitical context’, comprising governance, policies and wider societal norms (see Figure 1).

While reports [11,12,20] and scarce published research [21] indicate that HMPs ever more address issues also beyond healthcare access (e.g., knowledge gaps, discrimination, socioeconomic issues), scientific studies comprehensively evaluating the agenda and practice of HMPs with respect to SDH are lacking. We therefore performed a qualitative evaluation study focused on these aspects of one of the national HMPs, the ‘Healthy Communities’ program based in Slovakia. The program seemed especially appropriate for such an evaluation, as it ranks among the oldest and largest HMPs in the region and declares an emphasis on health-education rather than on healthcare access facilitation [22]. Our study aimed to evaluate how SDH were addressed in the agenda and in the everyday implementation of the HMP. In turn, our study simultaneously informs contemporary theoretical SDH frameworks about their practical utility specifically regarding interventions aimed at alleviating ethnic health-disparities.

## 2. Materials and Methods

### 2.1. Setting and Sample

During the beginning of the study (May 2014), an NGO owned and ran the evaluated HMP, which at that time covered 144 out of the approximately 800 concentrated Roma enclaves in Slovakia [23]. The HMP was undergoing an expansion into new localities, negotiations for more substantial state support, and internal re-assessments of previous criticisms [11]. The management of the HMP approached the first author due to his previous ethnographic experience among the program recipients [24] to design and perform a critical qualitative evaluation, which would help identify the program’s internal limitations and potentials with respect to positive health effects and to fair power-relations with the targeted Roma communities (we devoted a parallel study to the second aim). In return, the management agreed by written contract to allow the authors independent use of the study data. In the last stage of the study (October 2015), the HMP was co-run by the Ministry of Health and covered 234 segregated localities.

Our final sample consisted of over 70 HMP participants from across the organizational levels and over 30 HMP recipients from the communities served. The organizational structure of the HMP during the study period and the structure of the final sample are summarized in Table 1.

### 2.2. Measures and Procedure

Our evaluation strategy was qualitative: to evaluate the practice of interest we relied primarily on data on the involved actors’ knowledge and perspectives as constructed using qualitative research methods [25]. We used the following ethnographic research techniques: long-term personal rapport-building, direct observation, informal in-depth elicitation, field-notes taking, semi-structured interviewing, follow-up interviewing and reading of related documentation [26,27]. As a result, our data consisted of: field-notes on direct observations, on informal elicitations, and on readings of documentation; audio recordings of interviews; and the original HMP program documentation (see Appendix A).

The first author performed all the fieldwork in two stages (see Figure 1). In Phase 1 (May–November 2014), after reading the HMP documentation, he used the first four ethnographic techniques without any intended focus on the study evaluation aims or theory. This was to discourage schematic accounts of the program and to acquire instead data on the place and significance of the program in the respondents’ everyday lives. He job-shadowed several managers and a new group of the HMP fieldworkers in one county (i.e., consulting and observing everybody performing all their main duties), and independently visited the communities the fieldworker group served. In addition, he regularly engaged in occasional observations of other HMP staff and recipients (e.g., at regular regional HMP training sessions and visiting recipient communities). To earn trust, along with everyday reciprocity, he engaged in open discussions with the respondents on their personal aspirations and mutual power relations (i.e., regarding different respondents themselves as well as regarding different respondents and himself).

In Phase 2 (December 2014–October 2015), the first author focused on collecting data according to the WHO SDH Framework (Figure 1) and related literature [18,28,29]. This was to obtain direct and complete data on how the HMP agenda and the everyday implementation addressed SDH. During this phase, the author regularly used unarranged and arranged meetings across the HMP organizational levels and recipient communities (e.g., meetings with the HMP management devoted to other purposes and planned interviews with coordinators) to conduct semi-structured interviews (see Appendix B) and later follow-up interviews discussing the preliminary findings and dilemmas regarding the study questions. In this phase, the author also directly encouraged respondents’ critical feedback regarding the program including its power-relations (e.g., by acknowledging his own critical reflections of the limited extent to which the fieldworkers’ views were being consulted upon the HMP planning).

As anticipated in the introduction, we chose the WHO SDH Framework, i.e., an SDH theory external to the program, for evaluation of the HMP for two reasons. First, reports and research indicate that the range of SDH that the CEE HMPs address is gradually expanding. Second, the WHO SDH Framework presents an exceptionally well-established and comprehensive framework on SDH [28,30].

### 2.3. Analyses and Reporting

We analyzed the data using directed qualitative content analysis [31]. First, all types of field notes and records of elicitations were transcribed by the first author into digital text and merged into a single MAXQDA (VERBI Software-Consult-Sozialforschung GmbH, Berlin, Germany) database. He then added the HMP original documentation to the database. Subsequently, he coded all texts in the database for relevance to particular categories of the SDH Framework. Simultaneously, he used codes to distinguish between data on the HMP agenda and everyday implementation to enable a direct comparison between agenda and practice in the analysis and the reporting of results. He coded all text related to how the program was supposed to work, as “HMP agenda”, i.e., whether originating in the program documentation or from elicitations. This was because we were interested in the agenda both in its normative written form and how it was understood by the program participants. All other text, considered as relating to the program’s everyday implementation, was coded as “HMP practice”. Consequently, the first author created separate summaries covering the HMP agenda and everyday implementation through recursive abstraction, i.e., repeated reading and summative abstraction of text coded as relating to the same categories [32]. Upon summarizing, he focused on identification of dominant patterns in the evaluated aspects of the HMP according to the respondents’ and his own recorded assertions. He focused simultaneously on capturing eventual differences and conflicts in the recorded assertions between different sources, i.e., various respondent groups, the HMP documentation and his observations.

We used the WHO SDH Framework as our criterion to evaluate the merits of the HMP regarding SDH. We reviewed and assessed, separately for the HMP agenda and the HMP everyday implementation, to what extent were our summaries of the HMP consistent with the WHO SDH Framework’s main assumptions regarding what needs to be addressed to alleviate health inequities. We report the main identified (in)consistencies below as our main findings, each in juxtaposition with the WHO SDH Framework’s related main assumption.

### 2.4. Ethics Approval

The original academic institution overseeing the study (Department of General Anthropology, Faculty of Humanities, Charles University in Prague) did not require or issue ethical approvals for qualitative research at the time the field work started. The study was, however, reviewed retrospectively by the current ethical committee of the Czech Association for Social Anthropology which confirmed that the research was carried out in line with the Association’s ethical guidelines which also fits the Helsinki guideline.

## 3. Results

In Table 2, we summarise our main findings, i.e., the identified HMP’s (in)consistencies with the WHO SDH Framework’s main assumptions regarding what needs to be addressed to alleviate health inequities. The findings are presented separately for the HMPs agenda and the everyday implementation, with each group of findings further sorted according to the WHO SDH Framework’s original main categories of ‘Intermediary SDH’, ‘Structural SDH’ and ‘Socio-political context’ (see Figure 1 and the original WHO source for definitions [18]).

Below, in each paragraph we first repeat each of the main findings cited in Table 2 (italicized) and then we explain it. We specify in the explanations on whose perspectives the finding has been based. In general, regarding the evaluated aspects we found no significant differences or conflicts in assertions depending on the source. Where particular findings were supported by only some types of sources, the other types of sources provided no inputs on that issue or were not relevant by definition (e.g., normative documentation with respect to everyday implementation). In both parts (i.e., parts covering the HMP agenda and the HMP everyday implementation), each descriptive section devoted to a specific SDH level concludes with a statement summarizing the section’s findings in terms of what they suggest regarding the merits of the HMP’s agenda or everyday implementation according to the WHO SDH Framework.

### 3.1. The HMP Agenda

#### 3.1.1. Intermediary SDH

*Most intermediary SDH were not supposed to be addressed, except for health-related behaviours and healthcare access.* In the program documentation, no goals, targets or procedures focused on material circumstances, psychosocial factors or social cohesion. Both in the HMP staff’s accounts and in related documentation, the HMP primary goal was “to increase the targeted individuals’ health-related knowledge, motivation and active engagement” through sustained ad hoc face-to-face “health edification” and occasional public educational events. Where requested by either the HMP recipients or healthcare providers, the assistants were also supposed to personally “support access of the communities to healthcare services” via personal facilitation of access for individuals.

*The program’s declared secondary goal, i.e., to facilitate healthcare access, was operationalized more precisely than, and in a way logically contradicting, the program’s declared primary goal, i.e., to educate regarding health-related behaviours*. Even though declaring it a secondary goal, the documentation and the participants described related targets and procedures more extensively and in more detail than the health education. Simultaneously, according to both sources, access-facilitation was supposed to be realized as an unconditioned continuous extra service. As admitted by the HMP staff across organizational levels during the follow-up discussions, the access-facilitation was thus logically set out to support rather than to challenge the recipients’ existing health-related behaviours.

Regarding intermediary SDH, in its agenda the HMP was thus *partially consistent* with the WHO SDH Framework’s recommendations.

#### 3.1.2. Structural SDH

*Social positions of the program recipients were not supposed to be addressed systematically.* In the later stages of the research, “capacity building” through “an increase in education and employment” emerged as an additional goal both in the documentation and in the managers’ utterances. The management described and put this goal forward as related to the structural SDH. However, as acknowledged by the managers in the follow-up interviews, this was “an exaggeration”. The capacity building was namely planned exclusively via continuation of employment and work-related training of the individual assistants and expansion of the HMP to new localities.

Regarding structural SDH, in its agenda the HMP was thus *inconsistent* with the WHO SDH Framework’s recommendations.

#### 3.1.3. Socio-Political Context

*Socio-political context was not supposed to be addressed.* Neither in the HMP documentation nor in anybody’s understanding was the HMP to advocate or to provide any systematic feedback regarding governance, policies or any societal and cultural norms and values ultimately affecting the segregated Roma communities.

Regarding socio-political context, in its agenda the HMP was thus *inconsistent* with the WHO SDH Framework’s recommendations.

### 3.2. The HMP Everyday Implementation

#### 3.2.1. Intermediary SDH

*Of all the intermediary SDH, the HMP assistants were most active and successful regarding facilitation of healthcare access.* According to HMP staff across organizational levels, the fieldworkers were increasingly approached by the residents of the communities and the local healthcare providers for assistance with access. This was also apparent in new localities, where assistants typically faced an initial phase of distrust from both groups (e.g., they hesitated to accept any offered help, explicitly questioned the assistants’ possible hidden agendas). The assistants were active mainly with consultations regarding clinical processes (e.g., interpretation of documentation, home medication planning), facilitation of communication (e.g., accompanying to appointments, translation between Romani and Slovak) and first aid. In addition, they provided on demand logistic help to local public health authorities (e.g., facilitation of water source decontamination or of interventions in outbreaks of local epidemics).

*The HMP’s healthcare access facilitation supported rather than challenged the existing healthcare access-related and other health-related behaviours of some recipients*. All respondents typically found the assistance with access unproblematic and helpful. According to some consulted fieldworkers, however, some of the community residents started to regard this HMP service as their patient right paid for by the government. For example, the first author witnessed several cases of community residents asking the assistants “to do their duty’’ and bring them their medicine. The management considered this phenomenon “a growing problem that will need to be addressed” (female executive board member) and informally instructed the fieldworkers to curb such understanding ad hoc.

*Educational activities aiming at behavioural change were considered inappropriate by both HMP recipients and assistants and neglected or appropriated by the latter, except regarding child and maternal health.* According to the participants and recipients consulted, unsolicited face-to-face educational activities and nudging were especially viewed as disrespectful. Most of the long-term observed assistants admitted that, consequently, they neglected or appropriated this “embarrassing, typically non-Roma-like (gadžikano) part of the job” (female HMP assistant); e.g., they would over-report the numbers of their edification visits or only visit their own extended family households. The inappropriateness of edification was supposed to be due to its patronizing nature (“A proper grown up Romňi will never listen to another Romňi preaching!”, female recipient in a rural settlement) and its sometimes-unrealistic content. In contrast, everybody considered occasional public educational events to be interesting and sometimes also helpful. The managers seemed vaguely aware of such assistants’ appropriations but mostly considered them as temporary breaches which tend to disappear as the assistants earn more trust of the recipient community’s residents. Also, some managers confessed that the HMP leaders merely copied most of their health edification approach from a Slovak Public Health Agency HMP pilot (running in approximately 30 localities in 2008–2011) and declared it the HMP’s priority for rather tactical reasons—to avoid conflicts with clinical healthcare professionals “touchy about the program fieldworkers becoming understood as clinicians” (male operational manager).

*After earning the HMP recipients’ trust, many assistants were successful at inspiring changes in health-related behaviours and helping individuals to cope with their psychosocial struggles.* According to everybody consulted, after an initial period of mistrust most assistants typically started to acquire new personal relationships with other locals beyond their previous affiliations. For some of these new friends, the assistants were supposed to increasingly present surprisingly strong inspiration or personal role models as well as personal counsellors regarding psychosocial issues. In the first author’s personal experience, the HMP recipients often referred to the assistants as to “their nurses, despite being poor Roma like them”, and shared examples of how the assistants “have taught them to take care of their health properly” (female recipient community member); e.g., local women would start better complying with medical recommendations regarding preventative check-ups, infant diet, vaccination plans. The managers were aware of the growing psychosocial counselling demand and claimed to be “currently looking into how to better equip the assistants for it” (male operational manager).

*Most coordinators engaged in and some were successful at addressing the material-circumstances related issues at the community level.* According to all respondent groups, beyond their duties most coordinators thus also seemed to deal with: sewerage, water sources, public lighting, waste disposal, rodent control and disinfection. The management seemed to encourage such deliberate engagements of coordinators informally, mostly through ad hoc consultations. Given the lack of systematic support, their primary workload and the procedural complexity of most of the issues (e.g., requiring knowledge of related laws and varying negotiation styles), the success in such activities seemed to depend mostly on the personal knowledge, experience, wit and endurance of the individual coordinators.

Regarding intermediary SDH, in its everyday implementation the HMP was thus *consistent* with the WHO SDH Framework’s recommendations.

#### 3.2.2. Structural SDH

*Most coordinators engaged in and some were successful at addressing local issues related to income, occupation and education.* According to everybody consulted, such issues were typically raised by the assistants upon following individual clinical cases. With informal ad hoc support from the management and depending on individual capacities, the coordinators appeared sometimes to be successful at resolving e.g., indebtedness, loss of employment or unsuccessful admissions to regular elementary schools. Such resolutions were supposed to have a community level impact through encouraging and instructing other locals.

*Some fieldworkers were active and successful at increasing a particular community’s bridging and linking social capital.* Both in direct relation to their duties and beyond, some coordinators and assistants appeared systematically to work on building and sustaining informal networks of personal long-term cooperation with sympathetic representatives of varied institutions and nearby non-Roma residents. The management informally supported these activities and generally viewed them as, in the words of one HMP executive board member, “an unprecedentedly effective way of connecting the settlements with public resources independently of the often indifferent and sometimes also corrupt and outright racist local municipal authorities”.

Regarding structural SDH, in its everyday implementation the HMP was thus *partially consistent* with the WHO SDH Framework’s recommendations.

#### 3.2.3. Socio-Political Context

*There were no systematic feedback or advocacy activities directed outside the HMP beyond activities aimed at strengthening the HMP itself.* According to those directly involved and to the author’s personal observations, the central management actively lobbied the central state institutions both directly and through the use of mass media. These were, however, exclusively ad hoc efforts aimed at securing and increasing the institutions’ support of and involvement in the project itself (e.g., continuation of financial support provided by the Ministry of Health).

*The central management considered the HMP inadequate and too unstable to handle extensions with respect to the socio-political context.* All respondent groups acknowledged that they kept accumulating extensive experience and evidence regarding structural obstacles the HMP recipients faced, also at the societal level. For example, they regularly witnessed incidents of neglect and racism by healthcare providers or practical inappropriateness of legislative regulations. The managers did not contemplate any systematic use of this know-how for two reasons. First, they viewed issues related to the socio-political context as lying outside the HMP mandate by definition—since “it is only supposed to deal with health issues” (female executive board member). Second, they viewed the program as operating in a generally hostile institutional environment and feared any substantial extensions might undermine the unstable support of external and internal stakeholders. They feared field-workforce overload, escalation of competence conflicts and possible performance decline. In the words of an executive board member, most cooperating entities viewed the HMP as “already a luxury” and the program leaders were thus “better not taking the risk of becoming identified as [human rights] activists […] at least until the project earns more political leverage through its undeniable achievements” (male operational manager).

Regarding socio-political context, in its everyday implementation the HMP was thus *inconsistent* with the WHO SDH Framework’s recommendations.

## 4. Discussion

We evaluated how SDH were addressed in the agenda and in the everyday implementation of the ‘Healthy communities’ HMP in Slovakia. We found that in its *agenda*, i.e., both in its written form and how it was understood by participants, the HMP did not account for material circumstances, psychosocial factors, social cohesion, structural determinants of health and the socio-political context. The program’s declared secondary goal, i.e., to facilitate healthcare access, was set out more precisely than, and in a way logically contradicting, the program’s declared primary goal, i.e., to educate regarding health-related behaviours. In the HMP *everyday implementation*, healthcare access facilitation activities appeared to be effective, well received by the HMP recipients and assistants, and performed as set out by the latter. The opposite was true for most educational activities targeting health-related behaviours. The HMP fieldworkers seemed very proactive and sometimes effective at addressing other SDH domains beyond the HMP agenda: inducing desired behavioural changes as role models; resolving issues related to material circumstances, psychosocial factors and social position; and accumulating knowledge regarding systematic local impacts of the socio-political context. The HMP leaders supported such deliberate engagement only informally, considering the program inadequate and too unstable to handle any such extensions.

We found that with respect to known SDH the focus set out in the HMP’s agenda was rather narrow (see Table 1). Similarly, an Open Society Institute (OSI) report reviewing all the HMPs in the region identified all the following SDH among “what current Roma HMPs do not address”: income poverty, discrimination, health policy and legislation, and lack of resource commitment and political will [13]. The latest WHO report on the national HMP in Romania acknowledged the same [12]. Despite presenting the most consistent (and often the only) national policies implemented to alleviate the steepest health disparities in the CEE region, in their theories the HMPs thus seem to be similarly constrained to only a narrow section of the intermediary SDH.

We found that in the HMP agenda, healthcare access facilitation activities were logically set out to support rather than to challenge the program recipients’ existing healthcare access-related and other health-related behaviours. This corresponded with our finding that in the HMP’s everyday implementation, many recipients understood and used the healthcare facilitation services as continuous extra free healthcare service. The OSI [13] report found similar trends in all of the CEE HMPs: “paradoxically, mediation may serve to increase the distance between patient and doctor, and, unless the mediator seeks to educate the patient, may perpetuate the need for health mediators.” A follow-up report [11] adds: “[Mediators report that] physicians sometimes asked them to explain things to the patient, rather than the doctor trying to do so. There were isolated reports of Roma health mediators going to social service or doctor appointments without the client.” In their current setups and implementation, the CEE HMPs thus seem to hinder their own eventual health-promotion activities (certainly regarding healthcare use) by fostering dependence of the recipient communities on their services, i.e., supporting the medicalisation of communities [33].

We found poor acceptability and extensive appropriation of face-to-face educational activities and nudging targeting health-behaviours within the HMP. So far, very little attention has been paid directly to the educational activities within CEE HMPs elsewhere. Our finding, however, fits well with Schneeweis’s [21] unique and delicate account of how mediators themselves understand and manage their everyday health-mediation work in Romania. Based on our discussions with the managers, we identified two sets of circumstances possibly contributing to these phenomena. First, there seemed to be little to no methodological consultation involved on the part of the HMP planners with contemporary expertise in health promotion favouring participatory tailoring [34,35]. The conceptual framing and historical links we identified with respect to the formal priority of “health edification” within the HMP suggest it presents only a residue of the now nearly dismantled Communist public health system [36] and its paternalist approach to Roma [37,38]. Second, the ethnic framing of the poor acceptability put forward by the involved Roma points to the possibility, long discussed among anthropologists [3], of segregated Roma and analogous groups also constructing their ethnicity in direct contrast to local non-Roma norms. Analogous tensions and practices of appropriation in developmental programs targeting Roma in Romania are discussed in depth e.g., by Ivasiuc [39].

We found that many of the HMP fieldworkers spontaneously incorporated most of the SDH lacking in the HMP agenda (see Table 1) into their everyday implementation of the program. This finding corresponds with and significantly extends existing indices of similar positive potentials in most of the other HMPs [11,12,13,20]. In contrast with the other HMPs in CEE, the evaluated HMP seemed to exhibit unparalleled capacities especially with respect to the structural SDH, and this apparently was mainly through the HMP coordinator role (see Table 1). It thus seems that, importantly, while the fieldworkers in the CEE HMPs often deliberately appropriate their assigned duties, they typically do so to increase their own impact regarding SDH rather than solely for their private benefit. Also, the identified capability of the HMP fieldworkers to shift the actual intervention of the program successfully towards the more “upstream” SDH might present another indicator of the HMP leaders’ genuinely collaborative approach to participation (see also the low-threshold hiring criteria and managerial support regarding issues raised by assistants beyond the program agenda) [40,41]. Creating more capacity in the HMPs for addressing SDH more extensively, e.g., through strengthening of positions, such as those of the coordinators, could thus greatly improve the overall impact of the HMPs at least regarding local structural aspects. Further, even within their current capacity constraints the HMPs could increase their impact regarding more upstream, wider structural and societal determinants, by systematically collecting the HMP fieldworkers’ experiences with these factors and feeding this information to the respective responsible governing institutions.

We found that the HMP central management did not attempt to exploit the fieldworkers’ interest and capacities with respect to SDH mainly due to their viewing the institutional environment in which the HMP operated as generally hostile. This sadly fits with all of the above-cited reports’ long-term, and long-term ignored, key recommendations appealing to policy-makers to end the devastatingly precarious status of HMPs in CEE [11,12,13,20]. For analogous findings regarding community health-workers in general, see e.g., Lehmann and Sanders’s 2007 review [42].

In addition, we found that the decidedly negative attitude of central managements regarding the possibility of extending the HMP with SDH agendas devoted specifically to the socio-political context was based also on viewing such agendas as being beyond the realm of health. This finding matches the observations that health-promotion practice is everywhere still mostly conceived of according to the individual-level factors-rather than to the SDH paradigm [34,41,43]. To overcome these hindrances at the managerial level, greater institutional support of the HMPs by external actors and closer cooperation between the HMPs leaders and experts in public health thus seem indispensable.

Our study also provides a case for the utility of theoretical SDH frameworks in the design and evaluation of interventions aimed at alleviation of ethnic health disparities. For instance, our observations and elicitations structured according to the WHO SDH Framework have helped to identify the HMP fieldworkers’ and recipients’ experiences and views regarding specific influences of both wider socio-political conditions, as well as of local cultural idiosyncrasies linked to ethnicity. In line with current reviews in the field [44,45], our study thus confirms that coupled with open-ended qualitative approach, the biosocial theoretical frameworks used in state-of-the-art public health practice are sufficient for the identification of important mechanisms and pathways regarding health inequities even in historically and culturally rather complex situations.

### Strengths and Limitations

The key strength of our research dwelled in the long-term personal embeddedness of the first author within the examined HMP across its organizational levels. In addition, most of the long-term observations and elicitations took place in a geographical area where and among people with whom the first author has previously lived as an ethnographer [24]. These circumstances, in combination with our systematic encouragement of the respondents’ critical feedback, made it possible to obtain data of unusual depth, and robustness.

The main limitations of our research were due to the purposefully chosen qualitative strategy. Seeking well-grounded insights into the HMP’s everyday potentials via long-term personal embeddedness among only some of its participants and recipients, we could not deliver findings readily representative of the whole program. Also, for logistical reasons we did not manage to obtain data on perspectives of other actors involved in the HMP practice, such as representatives of local authorities or healthcare providers. Data collection, coding and underlying initial analyses were all performed exclusively by the first author. Lastly, we faced the tactics that respondents used to curb the imagined and real power asymmetries between themselves and other research participants (e.g., in all respondent groups we experienced initial distrust and follow-up adjustments of responses). The first author's long-term personal presence and encouragement of critical feedback might have limited some power-related biases. However, our exploratory findings do require further confirmation.

## 5. Conclusions

Unlike in its agenda, in its everyday implementation the evaluated HMP addressed most known SDH. Reports indicate that similar discrepancies between agenda and practice occur in other CEE HMPs. To increase the impact of HMPs on SDH, their theories and procedures should be adapted according to the programs’ more promising actual practice regarding SDH. To enable this, we advise closer cooperation between the HMPs’ leaders and public health experts and an increase of the HMPs’ institutional stability.

## Figures and Tables

**Table 1:** The organizational structure of the evaluated health-mediation program (HMP) and the structure of the final sample.

HMP Organizational Structure ^1^					HMP Recipients
	Fieldworkers		Central Management		
	Assistants	Coordinators	Management	Executive Board	
No. of persons	200	20	6	4	Approx. 60,000
Main duties	Community-based health education and facilitation of healthcare access	Support and supervision of the HM assistants	HMP operational management and public relations	Strategic decision-making, fund-raising and lobbying	N/A
Criteria for hiring	Completed elementary education; residency in the community of service & personal motivation (brief questionnaire)	completed secondary education; proficiency in Romani language; related previous experience and personal motivation (interview)	Previous related experience and personal motivation (interview)	N/A	N/A
Roma/non-Roma Ethnicity	Self-declared Roma, with few exceptions	Self-declared Roma, with one exception	Self-declared non-Roma, with one exception	Self-declared Non-Roma	Self-declared Roma
Approx. female: male ratio	3:1	1:1	2:1	1:1	1:1
Location of participants’ practice/target communities	Community-based, 1 per segregated settlement	Rotating visits of 10 assistants working in 1 area	In Bratislava	In Bratislava	Across the country, in 23 counties
**Final Sample Structure**					
Number of job-shadowed or long-term observed respondents/Study phase(s); Observation length per person	9 Phase 1; 1–3 weeks	1 Phase 1; 3 months	4 Phases 1 + 2; 3 to 14 days	0 N/A	18 Phase 1; 1–4 weeks
Number of occasionally observed and informally elicited respondents/Study phases	61 Phases 1 + 2	6 Phases 1 + 2	7 Phases 1 + 2	3 Phases 1 + 2	39 Phases 1 + 2
Number of persons who attended both structured and follow-up interviews/Study Phase	5 Phase 2	4 Phase 2	3 Phase 2	1 Phase 2	0 N/A
**Final Sample Size**	116 ^2^				

^1^ All numbers in this section are long-term averages (rounded where greater 10), as exact numbers fluctuated during the study period (see e.g., the exact numbers of localities in the main text); ^2^ Sum from the row ‘No. of occasionally observed and informally elicited respondents’—respondents enumerated in the other rows were people from this group.

**Table 2:** The health-mediation program’s (HMP) (in)consistencies with the World Health Organization’s Framework for action on social determinants of health (WHO SDH Framework) [18] ^1^.

SDH to Be Addressed According to the WHO SDH Framework	How Well Did the HMP Address the Recommended SDH?	
	In Its Agenda	In Its Everyday Implementation
**Intermediary determinants**	Most intermediary SDH were not supposed to be addressed, except for health-related behaviours and healthcare access The program’s declared secondary goal, i.e., to facilitate healthcare access, was operationalized more precisely than, and in a way logically contradicting, the program’s declared primary goal, i.e., to educate regarding health-related behaviours	Of all the intermediary SDH, the HMP assistants were most active and successful regarding facilitation of healthcare access The HMP’s healthcare access facilitation supported rather than challenged the existing healthcare access-related and other health-related behaviours of some recipients Educational activities aiming at behavioural change were considered inappropriate by both HMP recipients and assistants and neglected or appropriated by the latter, except regarding child and maternal health After earning the HMP recipients’ trust, many assistants were successful at inspiring changes in health-related behaviours and helping individuals to cope with their psychosocial struggles Most coordinators engaged in and some were successful at addressing the material-circumstances related issues at the community level
	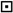	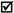
**Structural determinants**	Social positions of the program recipients were not supposed to be addressed systematically	Most coordinators engaged in and some were successful at addressing local issues related to income, occupation and education Some fieldworkers were active and successful at increasing a particular community’s bridging and linking social capital
	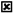	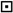
**Socio-political context**	Socio-political context was not supposed to be addressed	There were no systematic feedback or advocacy activities directed outside the HMP beyond activities aimed at strengthening the HMP itself The central management considered the HMP inadequate and too unstable to handle extensions with respect to socio-political context
	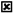	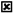

^1^ The symbol 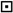 designates ‘partially’, 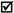 designates ‘well’, and 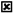 designates ‘poorly’; we use the symbols to summarize how well the HMP addressed each main category of SDH according to the listed main findings.

## Appendix A

**The original evaluated health-mediation program’s documentation included in the data for analysis (The year(s) between the brackets indicate particular version(s) included)**

**Descriptions of the program agenda and practice** Project Healthy Communities—Description of the Healthy Communities project [*Projekt Zdravé komunity-Popis projektu Zdravé komunity*] (2013) Healthy Communities—Final report [*Zdravé komunity Záverečná správa*] (2014) The statute of the association of legal entities Platform for promotion of the health of disadvantaged groups [*Stanovy občianskeho združenia Platforma pre podporu zdravia znevýhodnených skupín*] (2014) National project ‘Healthy Communities’ proposal [*Zámer národného projektu Zdravé komunity*] (2014, 2015) Project Healthy Communities Application Appendix 1—Project description [Žiadosť o poskytnutie nenávratného finančného príspevku Príloha 1—Opis projektu] (2014, 2015) Job specification for the Health edification assistant role [*Pracovná náplň Asistenta osvety zdravia*] (2014) Job specification for the Coordinator of the Health edification assistants role [*Pracovná náplň Koordinátora asistentov zdravotnej osvety*] (2014) Health edification assistant role activity sheets [*Pracovné výkazy asistentov osvety zdravia*] (2014, 2015) **Recruitment forms** Health edification assistant questionnaire [*Dotazník pre asistentov/ky osvety zdravia*] (2014) Coordinator of the Health edification assistants questionnaire [*Dotazník pre koordinátorov asistentov osvety zdravia*] (2014) Curriculum form [*Formulár Životopis*] (2014) **Training documentation and materials** The health edification assistant role [*Úloha asistenta zdravotnej osvety*] (2014) Care for pregnant women and new-borns [*Starostlivosť o tehotné ženy a novorodencov*] (2014) Human biology basics [*Základy biológie človeka*] (2014) Epidemiology [*Epidemiológia*] (2014) Basic communication skills for work within the Roma communities [*Základné komunikačné zručnosti v práci s rómskymi komunitami*] (2014) Specialized social counselling with respect to health care [*Špecializované sociálne poradenstvo v oblasti zdravotníctva*] (2014)

## Appendix B

**Semi-structured interviews outline**

***The program participation in the context of the participant’s life*** How did your life change after you took up your position within the program (explain the positives and negatives)? What do you like/don’t like about your current job? ***Personal account of segregated Roma health status*** Do you think there are differences in health between the Roma and the non-Roma? Based on what? If so, where do you think these differences come from? Based on what? What do you think could be done in order to alleviate these differences? Based on what? ***Personal account of the program agenda*** Why did the program start and who started it? Using what money? Where do you know this from? What does the program management claim it wants to achieve? What do you think the program management wants to achieve in reality? Where do you know these things from? How do you think the management wants to achieve these things? Where do you know this from? Do you think the program should focus on and should be doing something else as well? Why? Do you think enough attention is paid in the program set-up to whatever damages people’s health directly (e.g., material conditions, circumstances causing stress, risky health behaviours, specific bodily characteristics, and access to healthcare)? How? Do you think enough attention is paid in the program set-up to whatever else might be contributing to the worse health in the segregated communities (e.g., education, occupation, income, gender roles, and incidents of racism)? How? Do you think the program is well set-up to positively influence whatever might be affecting the health in the segregated communities at the country level (e.g., how these issues are governed centrally, particular related policies, the wide-spread anti-Roma racism) How? ***Personal account of the program practice*** Which of its goals is the program successful at achieving? How come? Based on what do you think that? Which of its goals is the program unsuccessful at achieving? How come? Based on what do you think that? Do you think the program is successful at dealing with whatever damages people’s health directly (e.g., material conditions, circumstances causing stress, risky health behaviours, specific bodily characteristics, and access to healthcare)? In what in particular? Based on what do you think that? What else should be done in this area and what should be done differently? Why? Do you think the program is successful at dealing with whatever else might be contributing to the worse health in the segregated communities (e.g., education, occupation, income, gender roles, and incidents of racism)? In what in particular? Based on what do you think that? What else should be done in this area and what should be done differently? Why? Do you think the program is successful at positively influencing whatever might be affecting the health in the segregated communities at the country level (e.g., how these issues are governed centrally, particular related policies, the wide-spread anti-Roma racism)? Based on what do you think that? What else should be done in this area and what should be done differently? Why?
